# Lychee Seed Fraction Inhibits Aβ(1-42)-Induced Neuroinflammation in BV-2 Cells via NF-κB Signaling Pathway

**DOI:** 10.3389/fphar.2018.00380

**Published:** 2018-04-23

**Authors:** Ya Zhao, Yuan Zeng, Anguo Wu, Chonglin Yu, Yong Tang, Xiuling Wang, Rui Xiong, Haixia Chen, Jianming Wu, Dalian Qin

**Affiliations:** ^1^Laboratory of Chinese Materia Medica, Department of Pharmacology, School of Pharmacy, Southwest Medical University, Luzhou, China; ^2^Department of Pharmacy, Affiliated Hospital of Southwest Medical University, Luzhou, China; ^3^Department of Human Anatomy, School of Preclinical Medicine, Southwest Medical University, Luzhou, China

**Keywords:** lychee seed, Alzheimer’s disease, anti-neuroinflammation, Aβ(1-42), NF-κB, Bcl-2, Bax

## Abstract

In our previous studies, an active fraction derived from lychee seed could inhibit β-amyloid-induced apoptosis of PC12 cells and neurons. The primarily microglia cells are recognized as the brain’s resident macrophages and thought to remodel of the brain by removing presumably redundant, apoptotic neurons. In the current study, we aimed to investigate the anti-neuroinflammation effect of lychee seed fraction (LSF) in Aβ(1-42)-induced BV-2 cells and the underlying mechanism. The morphology results displayed that LSF could improve the status of Aβ(1-42)-induced BV-2 cells. The enzyme-linked immunosorbent assay, real-time PCR, and Western blotting results showed that LSF could significantly reduce the release, mRNA levels, and protein expressions of the pro-inflammatory cytokines such as interleukin-1β (IL-1β), tumor necrosis factor alpha (TNF-α), cyclooxygenase-2 (COX-2), and inducible nitric oxide synthase (iNOS) in Aβ(1-42)-induced BV-2 cells, which were downregulated through suppressing the NF-κB signaling pathway. Furthermore, LSF could upregulate Bcl-2 and downregulate Bax, Caspase-3, and cleaved-PARP protein expressions. Taken together, our results first demonstrated that LSF could suppress the inflammatory response via inhibiting NF-κB signaling pathway, and inhibit apoptosis in Aβ(1-42)-induced BV-2 cells. Our findings further prove that LSF as a potential drug may be used for treating AD in the future.

## Introduction

Alzheimer’s disease (AD) is a chronic and progressive neurodegenerative disorder and it is characterized clinically with dysfunction in cognitive and behavior (Chen et al., 2014). The three main pathological features in AD patients’ brain are senile plaques (SP), neurofibrillary tangles (NFTs), and cerebral cortex atrophy (Carr, 2015; Akhtar et al., 2016; Li et al., 2016). As it is known, Aβ is the core of SP and which can accelerate the neuroinflammatory process. The excessive generated and deposited Aβ will initiate the AD pathological cascade, and lead to neuronal dysfunction and memory impairment (Huang et al., 2012). Microglia, the resident immune cells in the central nervous system (CNS), play an important role in the brain’s innate immunity and inflammatory neuropathologies (Nelson et al., 2002). BV-2 cells are derived from raf/myc-immortalized murine neonatal microglia and express morphological, phenotypical, and functional markers of macrophages (Haw et al., 2014). With respect to neurodegeneration studies, BV-2 cells were used in more than hundreds of publications as the neuroinflammation cell model. Therefore, in this current study, Aβ(1-42)-induced BV-2 cells were used to simulate the neuroinflammation in AD patient brain.

Emerging evidence suggests that Aβ plays an important role in the induction of neuroinflammation in AD (Stewart et al., 1997; McGeer and McGeer, 1998; Ledford, 2010). The extracellular deposited and intracellular generated Aβ can activate the astrocytes and microglia cells (Origlia et al., 2014), which then release amounts of the pro-inflammatory cytokines such as tumor necrosis factor alpha (TNF-α), interleukin-1β (IL-1β), cyclooxygenase-2 (COX-2), and inducible nitric oxide synthase (iNOS) (Weiner et al., 2012). These cytokines not only directly damage neurons (Fiala and Veerhuis, 2010; Guo et al., 2015), but also can promote the development of AD via boosting Aβ deposition and neuronal apoptosis. Nuclear factor-κB (NF-κB) as a pivotal mediator of inflammatory responses regulates the release and expressions of the pro-inflammatory cytokines such as TNF-α, IL-1β, COX-2, and iNOS. It was reported that the activation of NF-κB signaling pathway could result in the secretion of pro-inflammatory cytokines, which further promoted the degeneration of neurons in AD patients (Ulivi et al., 2008; Takata et al., 2011). Therefore, suppression of neuroinflammation via inhibition of NF-κB signaling pathway can ameliorate neurodegeneration and memory impairment (Asanuma et al., 2004), which may be a beneficial strategy for AD treatment.

Chinese medicinal herbs (CMHs) have been used to treat neurodegenerative diseases for a long time. Recently, many chemical components or extracts derived from CMHs were reported to modulate the neurodegeneration (Wu et al., 2013, 2015, 2017; Liu J.F. et al., 2015; Liu Y.M. et al., 2015; Wong et al., 2015; Xie et al., 2015; Yaidikar and Thakur, 2015; Jesky and Chen, 2016; Meng et al., 2016; Dong et al., 2017). Lychee seed, a famous traditional Chinese medicine, was recorded in “Ben Cao Gang Mu.” It was reported to exert a variety of bioactivities including anti-oxidant, anti-obesity, anti-viral, anti-tyrosinase, anti-microbial, anti-apoptotic, etc., (Prasad et al., 2009; Shahwar et al., 2010; Bhoopat et al., 2011; Qi et al., 2015). Our previous study first showed that the fraction derived from lychee seed fraction (LSF) could improve the cognitive function and prevent neuronal injury in AD rat and inhibit Aβ(25-35)-induced apoptosis in PC12 cells through NF-κB signaling pathway (Wang et al., 2017a,b). In the current study, we have investigated the anti-neuroinflammation of LSF in Aβ(1-42)-induced BV-2 cells using enzyme-linked immunosorbent assay (ELISA), real-time PCR (RT-PCR), and Western blotting methods. LSF was found to significantly reduce the release, mRNA levels, and protein expressions of the pro-inflammatory mediators such as TNF-α, IL-1β, COX-2, and iNOS, which were regulated via NF-κB signaling pathway. Furthermore, LSF could inhibit apoptosis in Aβ(1-42)-induced BV-2 cells by upregulating Bcl-2 and downregulating Bax, Caspase-3, and cleaved-PARP protein expressions. Therefore, this study provides a detailed insight into the neuroprotective mechanism and therapeutic application of LSF, which is valuable for further investigation as a novel drug for the treatment of AD.

## Materials and Methods

### Reagents

Aβ(1-42) was obtained from Sigma-Aldrich (St. Louis, MO, United States). Advanced RPMI 1640 medium (Gibco), fetal bovine serum (FBS), and RT-PCR kit were purchased from TransGen Biotech Limited Company (Beijing, China). Penicillin–streptomycin solution (100X), trypsin–EDTA solution, SDS–PAGE sample-loading buffer (5X), cell counting kit-8, primary antibody dilution buffer, secondary antibody dilution buffer, phenylmethanesulfonyl fluoride (PMSF), RIPA lysis buffer, and β-actin mouse monoclonal antibody were obtained from Beyotime Institute of Biotechnology (Shanghai, China). Fast Wright-Giemsa’s stain was obtained from Nanjing Jiancheng Bioengineering Institute (Nanjing, China). The primary antibodies such as NF-κB, IκBα, p-IκBα, Bax, Bcl-2, IL-1β, TNF-α, COX-2, and iNOS were purchased from Cell Signaling Technology, Inc. (Beverly, MA, United States). Caspase-3, cleaved-PARP and PARP antibodies were obtained from abcam (Cambridge, MA, United States). Primers used for RT-PCR analysis were synthesized by Invitrogen (Minato, Tokyo, Japan).

### Preparation of LSF

Lychee seed (origin: Luzhou, Sichuan) were obtained from the local herbal medicine market and authenticated by Professor Can Tang in Southwest Medical University. LSF was prepared according to the previous method (Wang et al., 2017a). In brief, 1 kg air-dried lychee seed were ground and soaked with 1 L 70% ethanol overnight, then extracted by percolation with 8 L 70% ethanol (8000 mL). Total 8.37 L percolation solution were collected and evaporated under vacuum. The dried collection dissolved with water was loaded onto D101 macroporous resins column and eluted with 70% ethanol. To the end, the eluted fraction yielded 31.75 g dried brown powder.

### Cell Culture

Primary cortical neurons BV-2 (a murine microglia cell line) cells were purchased from Conservation Genetics Chinese Academy of Sciences Kunming Cell Bank, which were cultured in RPMI 1640 medium supplemented with 20% fetal bovine serum, 50 U/mL penicillin, and 50 μg/mL streptomycin (Invitrogen, Scotland, United Kingdom) in a 5% humidified CO_2_ incubator at 37°C.

### Cell Viability

The LSF was dissolved with dimethyl sulfoxide (DMSO) to make a final concentration of 240 g/L. Cell viability of LSF against BV-2 cells was measured with CCK-8 according to the manufacture’s protocol. In brief, BV-2 cells were plated on a 96-well plate (100 μL, 1 × 10^5^ cells/well) 1 day before LSF treatment under the indicated concentrations. After 48 h treatment, 10 μL of CCK-8 solution was added into each well and incubated for another 2 h. Colorimetric reading of the solute mixture was then determined at OD 450 nm using a standard plate-reader (DG5032, Hua Dong, Nanjing, China). The percentage of cell viability was calculated using the following formula: Cell viability (%) = Cells number_treated_/Cells number_DMSO control_ × 100. Data were obtained from three independent experiments.

### Wright-Giemsa’s Staining

BV-2 cells were seeded on coverslips in a 6-well plate. The second day, the cells were pretreated with 5 μM Aβ(1-42) for 12 h and followed with an incubation of 0.12–0.48 mg⋅L^-1^ LSF for another 12 h. After treatment, these coverslips were air dried and stained with Giemsa solution as described previously (Chhabra et al., 2017). Briefly, slides were stained in the diluted Giemsa solution (1:20) in PBS for 15–20 min and washed with PBS to remove the excess stain, then the air-dried slides were observed and the optical microscope images were taken with an optical microscope (Leica DM750 optical microscope).

### Cytokines ELISA

BV-2 cells were plated in a 96-well plate (100 μL, 1 × 10^5^ cells/well) and pretreated with 5 μM Aβ(1-42) for 12 h, then followed with an incubation of 0.12–0.48 mg⋅L^-1^ LSF for another 12 h. The cell-free supernatants were subsequently employed for TNF-α, IL-1β, COX-2, and iNOS assays using the ELISA kits according to the manufacturer’s instructions.

### Real-Time PCR

In brief, BV-2 cells were seeded on a 6-well plate and pretreated with 5 μM Aβ(1-42) for 12 h, then followed with an incubation of 0.12–0.48 mg⋅L^-1^ LSF for another 12 h. After treatment, total RNA was extracted from BV-2 cells by using the Trizol reagent (Trans Serum, China) and its reverse transcription into cDNA was performed using a Prime Script RT reagent kit (Trans Serum, China) in accordance with the manufacture’s protocol. The primers used in this experiment are as follows:

**Table:** 

IL-1β:	Forward, CTAGGGACTTAGGTGCTGTC
	Reverse, CTCTGCCTTTGCTTCCAAGC
COX-2:	Forward, GATGACTGCCCAACTCCC
	Reverse, AACCCAGGTCCTCGCTTA
iNOS:	Forward, CGTTGGATTTGGAGCAGAAG
	Reverse, CCTCTTTCAGGTCACTTTGG
TNF-α:	Forward, GAGCACAGAAAGCATGATCC
	Reverse, GAGAAGAGGCTGAGACATAG
GAPDH:	Forward, ACCACAGTCCATGCCATCAC
	Reverse, TCCACCACCCTGTTGCTGTA.

### Western Blotting

After treatment, BV-2 cells were harvested and lysed by using RIPA lysis buffer (Beyotime, China) according to the manufacturer’s instructions. Protein concentrations were measured by BCA kit (Beyotime, China), and equal amounts of protein (50 μg/well) were loaded onto SDS–PAGE. After electrophoresis, the proteins were transferred onto the polyvinylidene fluoride (PVDF) membranes, which were then blocked with 5% non-fat dried milk for 1 h. The membranes were incubated with the primary antibodies including β-actin, IL-1β, COX-2, TNF-α, iNOS, NF-αB, IαBα, p-IαBα, Bax, Bcl-2, Caspase-3, cleaved-PARP and PARP overnight at 4°C, then followed an incubation with horseradish peroxidase-conjugated secondary antibodies for 1 h. Finally, the protein expression bands were revealed by the ultra ECL Western Blotting Detection Reagent (4A Biotech Co., Ltd., China) and detected by the ChemiDoc MP Imaging System (Bio-Rad). Band intensity was quantified using Image J software and the ratio of the interest proteins to β-actin was calculated.

### Flow Cytometry

Cell apoptosis of BV-2 cells was measured by flow cytometry using the annexin V staining kit (BD Biosciences, San Jose, CA, United States). In brief, BV-2 cells were pretreated with 5 μM Aβ(1-42) for 12 h, then followed with an incubation of 0.12–0.48 mg⋅L^-1^ LSF. After treatment, BV-2 cells were analyzed by the NovoCyte Flow Cytometer Systems (ACEA Biosciences) using FITC-annexin V and propidium iodide staining (BD Biosciences, San Jose, CA, United States) according to the manufacturer’s instructions. Data acquisition and analysis were performed with NovoExpress software (ACEA Biosciences).

### Hoechst 33342 Staining

Cell apoptosis of BV-2 cells was also detected using the Hoechst 33342 assay kit (Beyotime Institute of Biotechnology, China). In brief, BV-2 cells seeded on coverslips in a 6-well plate were pretreated with 5 μM Aβ(1-42) for 12 h, then followed with an incubation of 0.12–0.48 mg⋅L^-1^ LSF for another 12 h. After treatment, BV-2 cells were fixed in freshly prepared 4% paraformaldehyde for 15 min and washed with PBS for three times, then incubated with 5 mg/L Hoechst 33342 staining solution for 5 min. After incubation, the air-dried slides were mounted with FluorSave^TM^ mounting media (Calbiochem, San Diego, CA, United States) and subjected to fluorescence microscopic analysis for observing the apoptosis under the microscope (Invitrogen EVOS FL Auto Cell Imaging System). The cells with condensed and fragmented nuclei were considered to be apoptosis.

### Statistical Analysis

All data are presented as mean ± SD and analyzed using GraphPad Prism 5.0 statistical analytical software. The difference was considered statistically significant as *P* < 0.05. One-way ANOVA followed by post-Tukey was applied for statistical analysis to compare all the different groups in the current study.

## Results

### Cytotoxicity of LSF

**FIGURE 1:**
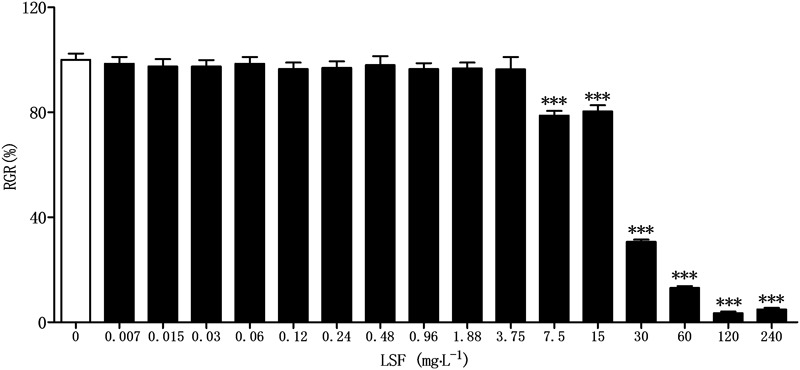
Cytotoxicity of LSF against BV-2 cells. BV-2 cells were treated with LSF (0.007–240 mg⋅L^-1^) for 48 h, and the relative growth rate (RGR) was calculated using CCK-8 method. ^∗∗∗^*P* < 0.001 vs. Control.

Before assaying the anti-neuroinflammation of LSF in Aβ(1-42)-induced BV-2 cells, CCK-8 method was applied to investigate the cytotoxicity of LSF against BV-2 cells. As showed in the **Figure 1**, there are no toxicity observed under the treatment of LSF with 3.75 mg⋅L^-1^.

### LSF Ameliorates the Abnormal Morphology of Aβ(1-42)-Induced BV-2 Cells

**FIGURE 2:**
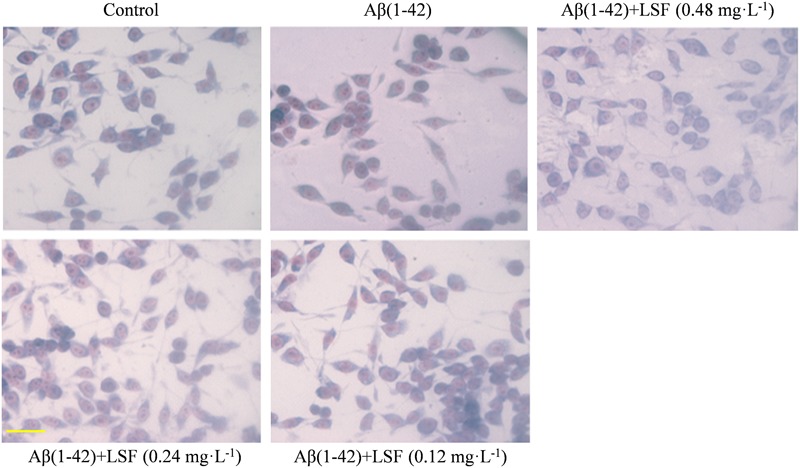
The morphological changes of BV-2 cells pretreated with 5 μM Aβ(1-42) for 12 h and followed with an incubation of 0.12–0.48 mg⋅L^-1^ LSF for another 12 h. Magnification: ×40; Scale bar: 15 μm.

The morphological changes of BV-2 cells treated with Aβ(1-42) or Aβ(1-42) and LSF (0.12–0.48 mg⋅L^-1^) were observed. The **Figure 2** showed that the cells treated with Aβ(1-42) (5 μM) alone displayed the fusiform, more synapse and obvious nucleus fragmentation, while LSF could improve the morphological status of BV-2 cells to some extent, which suggested that LSF might suppress the BV-2 activation induced by Aβ(1-42) and take protective effect in BV-2 cells.

### LSF Inhibits the Pro-Inflammatory Cytokines in Aβ(1-42)-Induced BV-2 Cells

**FIGURE 3:**
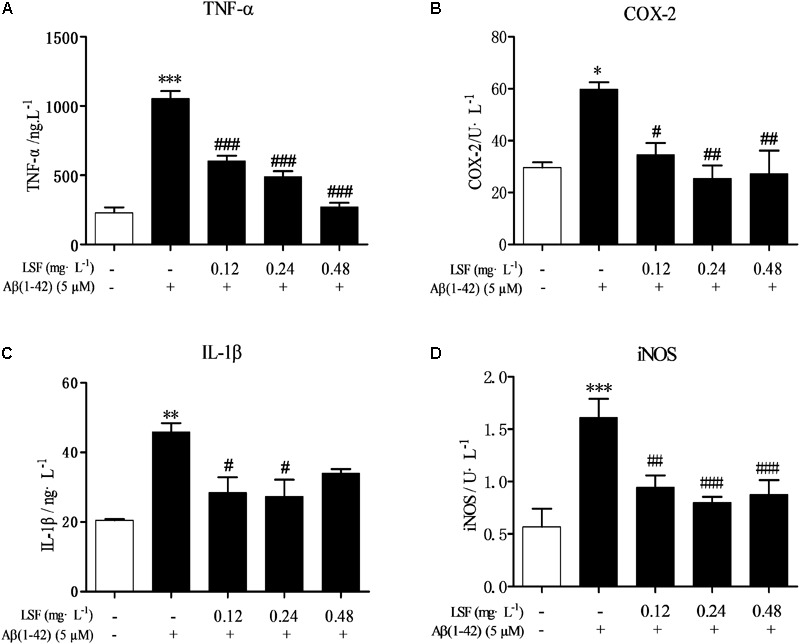
LSF reduces the release of the pro-inflammatory cytokines in Aβ(1-42)-induced BV-2 cells. After BV-2 cells were pretreated with 5 μM Aβ(1-42) for 12 h, then followed with an incubation of 0.12–0.48 mg⋅L^-1^ LSF for another 12 h. The cell-free supernatants were subsequently employed for TNF-α **(A)**, IL-1β **(B)**, COX-2 **(C)**, and iNOS **(D)** assays using ELISA kit. ^∗^*P* < 0.05, ^∗∗^*P* < 0.01, ^∗∗∗^*P* < 0.001 vs. Control; ^#^*P* < 0.05, ^##^*P* < 0.01, ^###^*P* < 0.001 vs. Model.

**FIGURE 4:**
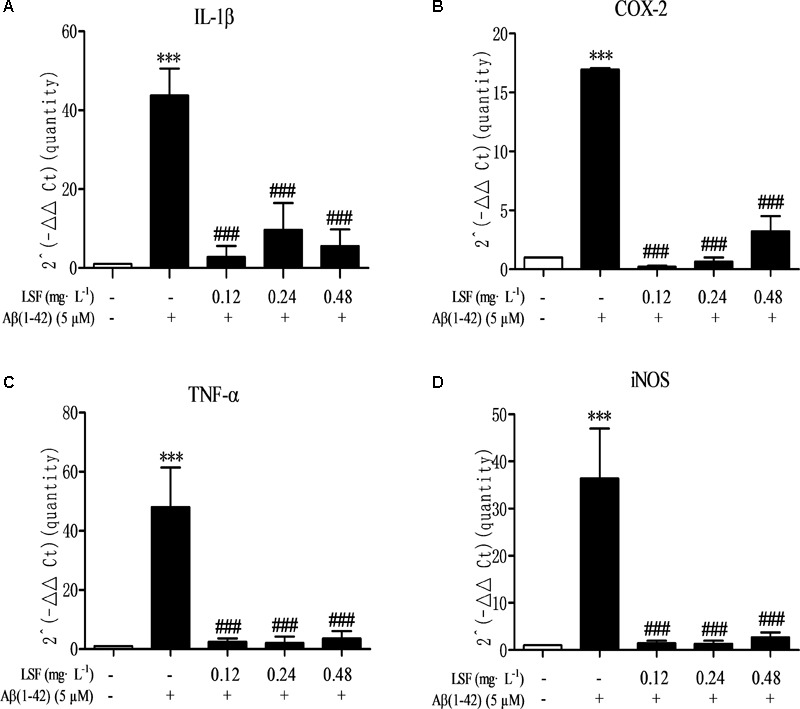
LSF decreases the mRNA levels of pro-inflammatory cytokines in Aβ(1-42)-induced BV-2 cells. The mRNA levels of TNF-α **(A)**, IL-1β **(B)**, COX-2 **(C)**, and iNOS **(D)** were measured by real-time PCR. GAPDH was used as an internal control. ^∗∗∗^*P* < 0.001 vs. Control; ^###^*P* < 0.001 vs. Model.

**FIGURE 5:**
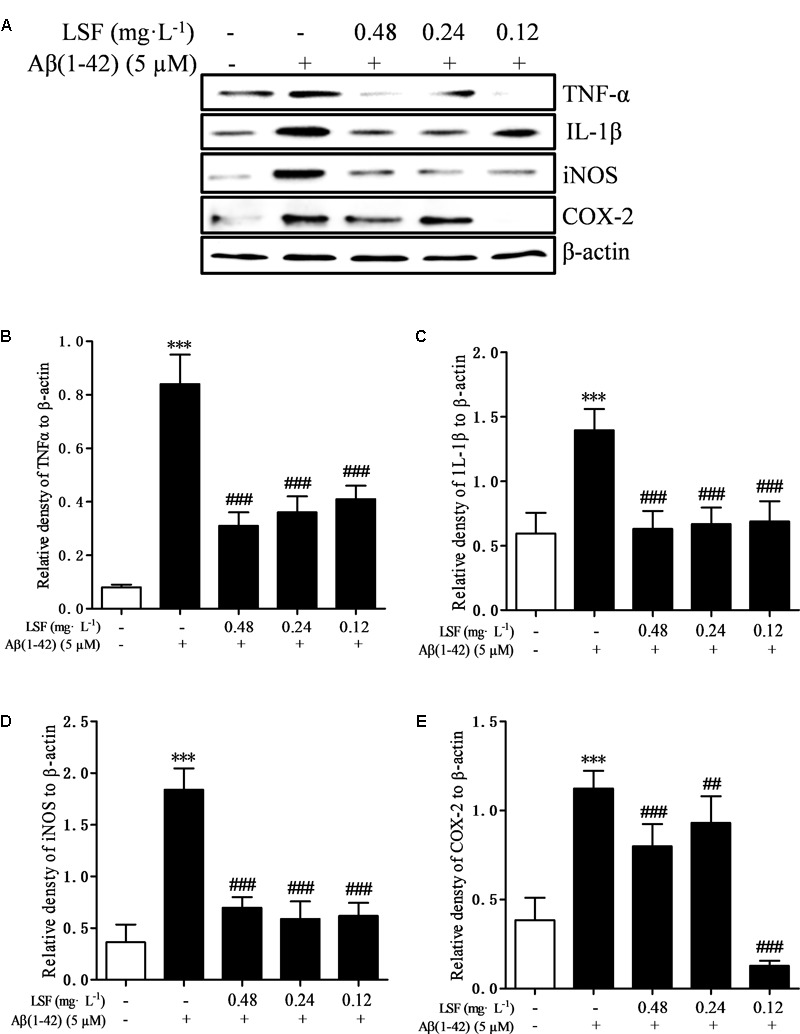
LSF reduces the protein expression of TNF-α, IL-1β, COX-2, and iNOS in Aβ(1-42)-induced BV-2 cells. BV-2 cells were pretreated with 5 μM Aβ(1-42) for 12 h, then followed with an incubation of 0.12–0.48 mg⋅L^-1^ LSF for another 12 h. Cell lysates were then harvested and analyzed for NF-κB p65, p-IκBα/IκBα, respectively **(A)**. Band intensities of NF-κB p65 **(B)** and p-IκBα/IκBα **(C)**, COX-2 **(D)**, and iNOS **(E)** were quantified using Image J software and normalized to β-actin. Bars are representatives of three independent experiments. ^∗∗∗^*P* < 0.001 vs. Control; ^##^*P* < 0.01, ^###^*P* < 0.001 vs. Model.

Microglia are considered to be pivotal players in innate immune and inflammatory responses in AD. As the microglial are over-activated, a wide range of inflammatory cytokines including ROS, IL-1β, IL-6, TNF-α, etc., release, and then induce neurons death. In the current study, we have first explored the optimal concentration of Aβ(1-42) with 5 μM could significantly promote the release of pro-inflammatory cytokines such as IL-1β, COX-2, and iNOS (Supplementary Figure 2), which were suppressed by the treatment of 0.12, 0.24, and 0.48 mg⋅L^-1^ LSF for 12 h treatment (Supplementary Figures 3, 4). Therefore, in the following experiments, we selected the optimal LSF concentration and treatment time to further investigate the inhibition effect of LSF in the release of the pro-inflammatory cytokines in Aβ(1-42)-induced BV-2 cells using ELISA. As showed in the **Figure 3**, LSF could significantly reduce the release of TNF-α, IL-1β, COX-2, and iNOS. In addition, LSF could significantly inhibit the mRNA levels of TNF-α, IL-1β, COX-2, and iNOS in Aβ(1-42)-induced BV-2 cells by using RT-PCR (**Figure 4**). Meanwhile, the Western blotting results demonstrated that LSF inhibited the protein expressions of TNF-α, IL-1β, COX-2, and iNOS in Aβ(1-42)-induced BV-2 cells (**Figure 5**). Taken together, LSF takes neuroprotective effect may via inhibiting the release, mRNA levels, and protein expressions of the pro-inflammatory cytokines in Aβ(1-42)-induced BV-2 cells.

### LSF Inhibits Aβ(1-42)-Induced Activation of NF-κB Signaling Pathway in BV-2 Cells

**FIGURE 6:**
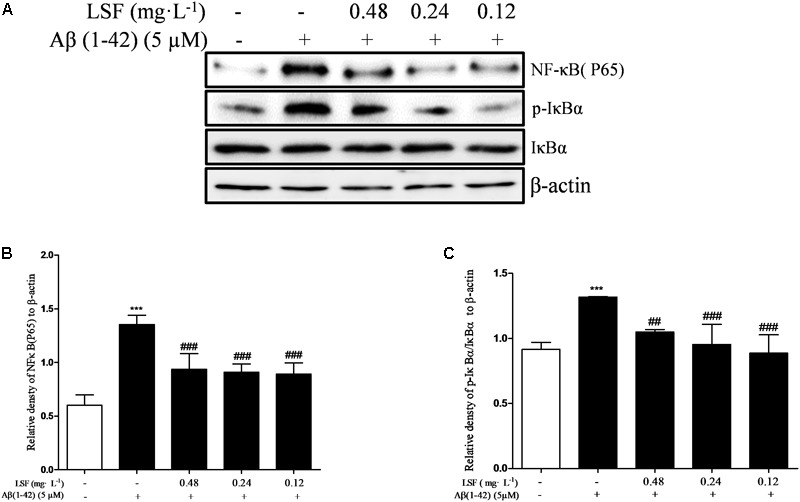
LSF inhibits the activation of NF-κB pathway in Aβ(1-42)-induced BV-2 cells. BV-2 cells were pretreated with 5 μM Aβ(1-42) for 12 h, then followed with an incubation of 0.12–0.48 mg⋅L^-1^ LSF for another 12 h. Cell lysates were then harvested and analyzed for NF-κB p65, IκBα/p-IκBα, respectively **(A)**. Band intensities of NF-κBp65 **(B)** and p-IκBβ/IκBβ **(C)** were quantified using Image J software and normalized to β-actin. Bars are representatives of three independent experiments. ^∗∗∗^ *P* < 0.001 vs. Control; ^##^*P* < 0.01, ^###^*P* < 0.001 vs. Model. The full-length blots are presented in Supplementary Figure 5.

NF-κB signaling pathway is the classic pathway in modulating inflammatory response in AD. Aβ(1-42) can activate NF-κB signaling pathway and release a variety of pro-inflammatory cytokines (Wang S.L. et al., 2014). In the above experiments, LSF has been proved to inhibit the release of pro-inflammatory cytokines. In this part, we have investigated the inhibition effect of LSF in regulating the NF-κB signaling pathway. As shown in **Figure 6**, LSF could significantly decrease the phosphorylation of IαBα and downregulate the NF-κB signaling pathway in Aβ(1-42)-induced BV-2 cells, which indicated that LSF suppressed the inflammatory response via inhibiting the NF-κB signaling pathway.

### LSF Inhibits Aβ(1-42)-Induced Apoptosis of BV-2 Cells

**FIGURE 7:**
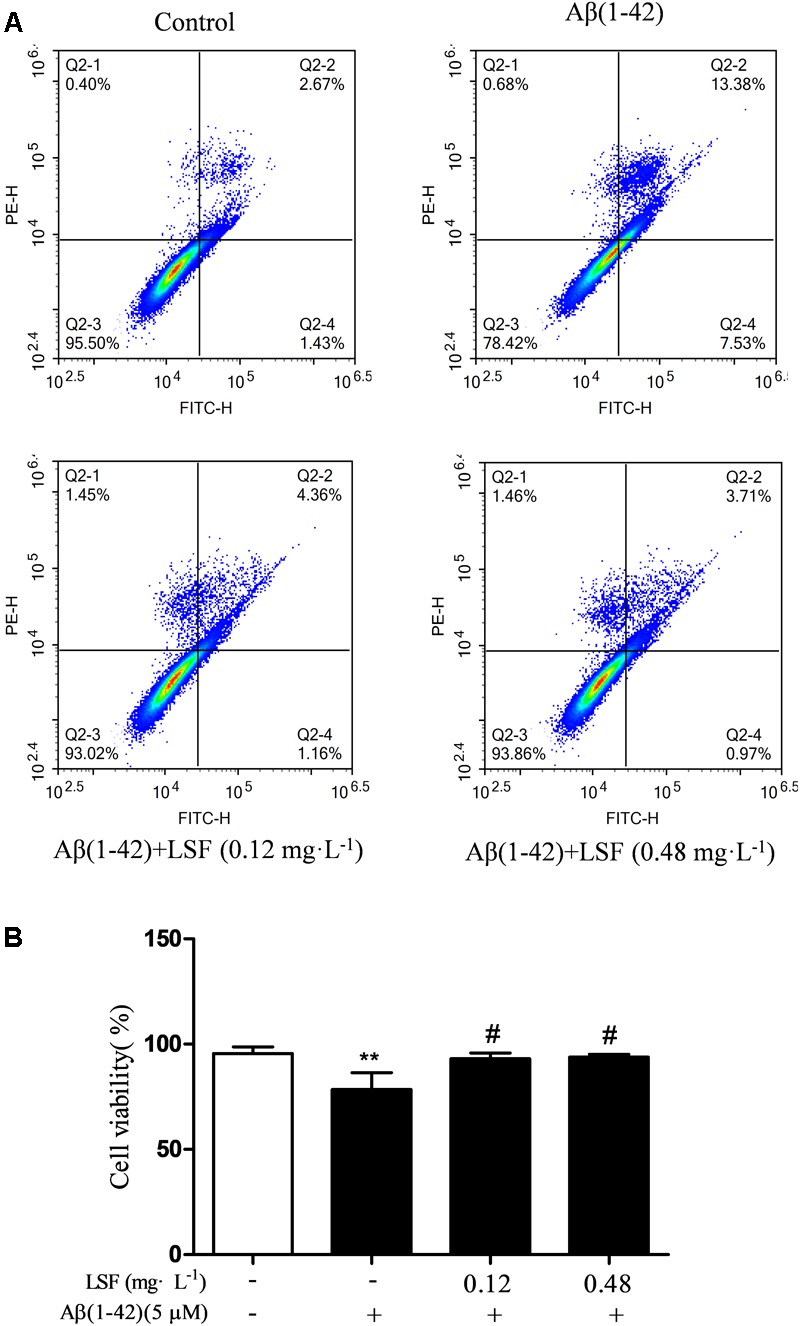
Flow cytometric analysis of the apoptosis in Aβ(1-42)-induced BV-2 cells. BV-2 cells were pretreated with 5 μM Aβ(1-42) for 12 h, then followed with an incubation of 0.12 and 0.48 mg⋅L^-1^ LSF for another 12 h. Representative flow cytometric analysis results showed the cell viability of BV-2 cells under these treatments **(A)**. Bar chart indicated the cell viability under these treatments **(B)**. Data from the flow cytometry analysis is represented as means ± SD of three independent experiments. Bars: SD. ^∗∗^*P* < 0.01 vs. Control, ^#^*P* < 0.01 vs. Model.

**FIGURE 8:**
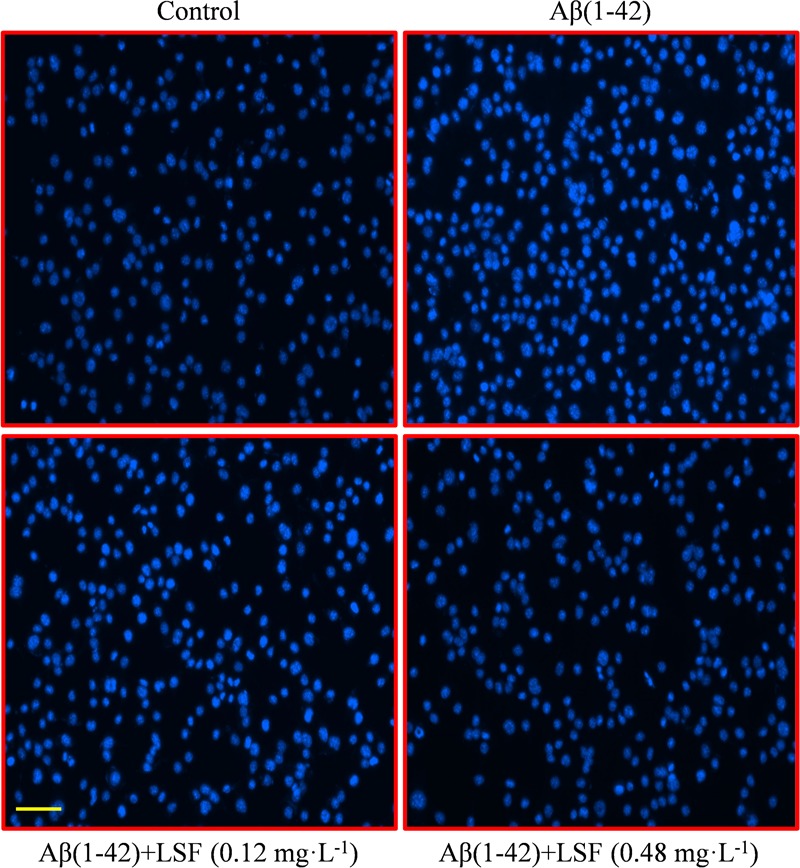
BV-2 cells apoptosis was observed using Hoechst 33342 staining. BV-2 cells were pretreated with 5 μM Aβ(1-42) for 12 h, then followed with an incubation of 0.12 and 0.48 mg⋅L^-1^ LSF for another 12 h. Apoptotic cells exhibited the morphological changes in the nuclei typical of apoptosis. Photos were taken under a fluorescence microscope (400×, magnification). Scale bar: 15 μm.

**FIGURE 9:**
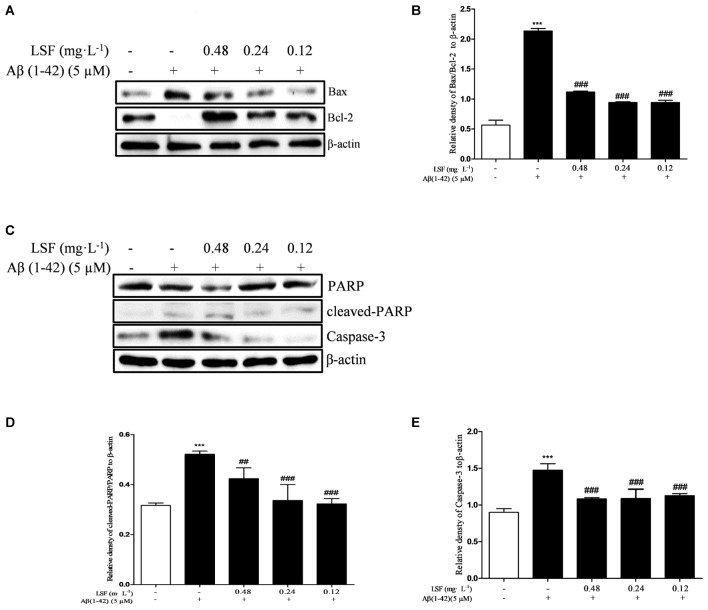
LSF attenuates apoptosis in Aβ(1-42)-induced BV-2 cells. BV-2 cells were pretreated with 5 μM Aβ(1-42) for 12 h, then followed with an incubation of 0.12–0.48 mg⋅L^-1^ LSF for another 12 h. Cell lysates were then harvested and analyzed for Bax/Bcl-2 **(A)**, Caspase-3 **(C)**, and cleaved-PARP/PARP **(C)**, respectively. Band intensities of Bax/Bcl-2 **(B)**, cleaved-PARP/PARP **(D)** and Caspase-3 **(E)** were quantified using Image J software and normalized to β-actin. Bars are representatives of three independent experiments. ^∗∗∗^*P* < 0.001 vs. Control; ^##^*P* < 0.01, ^###^*P* < 0.001 vs. Model. The full-length blots are presented in Supplementary Figure 5.

Emerging evidence suggests that Aβ(1-42)-induced over-activation of microglial can lead to microglial apoptosis and then result in uncontrolled inflammatory responses. In this part, flow cytometric analysis result demonstrated that LSF could significantly inhibit apoptosis in Aβ(1-42)-induced BV-2 cells (**Figure 7**), and the fluorescent microscopic analysis result which was obtained using Hoechst 33342 staining method displayed that LSF could reduce the cells with condensed and fragmented nuclei (**Figure 8**). Furthermore, LSF could upregulate Bcl-2 and downregulate Bax, Caspase-3, and cleaved-PARP protein expressions. Taken together, LSF could attenuate the apoptosis in Aβ(1-42)-induced BV-2 cells (**Figure 9**).

## Discussion

The LSF is an active fraction derived from lychee seed. In our previous studies, we have reported that the main components in this fraction were saponins. However, after analysis by using Agilent 6230 UHPLC-TOF-MS, the major components were actually proanthocyanidins (Supplementary Figure 1 and Supplementary Table 1) (Bhoopat et al., 2011; Wan et al., 2011; Su et al., 2014; Lv et al., 2015; Sui et al., 2016; Guo et al., 2017). Therefore, figuring out what are the detailed substances contributing to the bioactivity is essential.

Accumulated studies indicated that inflammation induced by Aβ is involved in neuronal degeneration in AD (Kitamura, 2011; Wullaert et al., 2011). Relevant reports showed that the levels of pro-inflammatory cytokines were significantly elevated in AD patients’ brain, which suggested that inflammation might contribute to the pathogenesis of AD (Ghosh and Karin, 2002). The extracellular deposited Aβ could activate glial cells and then release multiple inflammatory cytokines such as IL-6 and TNF-α, which promote neuronal damage (Hayden and Ghosh, 2008). Conversely, the injured neurons can release inflammatory factors, which can activate microglial to form a vicious circle to further aggravate the damage of neurons and promote the development of AD. Therefore, inhibiting the activation of glial cells and the production of pro-inflammatory cytokines may be a crucial approach to prevent and treat AD. In the present study, we have proved that LSF could significantly decrease the mRNA levels and protein expressions of IL-1β, TNF-α, COX-2, and iNOS in Aβ(1-42)-induced BV-2 cells.

**FIGURE 10:**
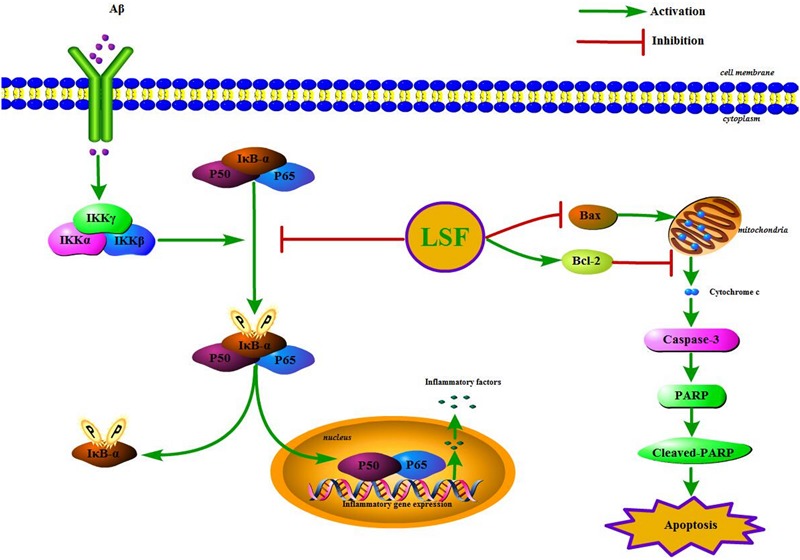
The IKK/IκBα/NF-κB signaling pathway and the apoptosis proteins are regulated by the treatment of LSF in Aβ(1-42)-induced BV-2 cells.

IκBα/NF-κB signaling pathway is reported to play an important role in modulating inflammatory response, and the suppression of NF-κB activation can ameliorate the neuroinflammation. In the resting cells, NF-κB family is composed of five members, p65 (RelA), RelB, c-Rel, p50/p105, and p52, which binds to IκBα and maintains NF-κB in an inactive form in the cytoplasm. Upon stimuli, IκBα is phosphorylated by IKK (IκB kinase) and NF-κB is translocated into the nucleus, which results in the release of the pro-inflammatory factors such as IL-1β, TNF-α, COX-2, and iNOS (**Figure 10**). This study first demonstrated that LSF could significantly inhibit the expression of NF-κB by attenuating Aβ(1-42)-induced IκBα phosphorylation and degradation *in vitro*, suggesting that LSF could suppress the pro-inflammatory cytokines via inhibiting NF-κB activation.

In our previous study, LSF was reported to inhibit apoptosis in Aβ(25-35)-induced PC12 cells, and also improve cognitive function and prevent neuronal injury in AD rats via regulating the apoptosis-related proteins. As is known to us, microglial activation is either neurotrophic or neurotoxic. The normally active microglia can response to neuronal damage and remove the damaged cells by phagocytosis and regulate inflammatory responses to pathogens, but the persistent-activated microglia can release the cytotoxins that result in neurotoxicity. At the same time, the over-activation can also lead to microglial apoptosis and then result in uncontrolled inflammatory responses. Therefore, the inhibition of the microglial cells apoptosis can inhibit the inflammatory response (Qin et al., 2012). The microglial apoptosis was reported to be regulated via multiple pathways including the activation of c-Jun N-terminal kinase and generation of specific reactive oxygen species, inhibition of the mitochondrial calcium uniporter, and PI3K-dependent signaling pathways (Kim and Li, 2013; Wang C. et al., 2014; Xie et al., 2017). In this study, the flow cytometric analysis showed that LSF suppressed the apoptosis rate of Aβ(1-42)-induced BV-2 cells. Meanwhile, LSF could also upregulate Bcl-2 and downregulate Bax, Caspase-3, cleaved-PARP and PARP protein expressions. However, the corresponding molecular mechanism is in need to be further investigated in the future.

Taken together, LSF could suppress the inflammatory response via regulating NF-κB pathway and inhibit apoptosis in Aβ(1-42)-induced BV-2 cells. However, the in-depth mechanism about the inhibition of the apoptosis in Aβ(1-42)-induced BV-2 cells, and especially in context with neuronal cells under the treatment of LSF are in need to elucidate with further studies. Therefore, these findings in this study provided evidences for LSF in anti-neuroinflammation effect *in vitro* and its further validation in transgenic AD animals is also essential in the future.

## Author Contributions

DQ and JW conceived and designed the experiments, contributed new reagents and analysis tools, and supervised all the research and revised the manuscript. YaZ, YuZ, AW, YT, XW, RX, HC, and CY performed the experiments. YaZ, YuZ, AW, JW, and DQ analyzed the data. YaZ, YuZ, and AW wrote the paper. All authors reviewed the manuscript.

## Conflict of Interest Statement

The authors declare that the research was conducted in the absence of any commercial or financial relationships that could be construed as a potential conflict of interest.
