# Supplementary material for: Lychee Seed Fraction Inhibits Aβ(1-42)-Induced Neuroinflammation in BV-2 Cells via NF-κB Signaling Pathway
Source: Front Pharmacol. 2018 Apr 23;9:380. doi: 10.3389/fphar.2018.00380 (PMC5925968; doi:10.3389/fphar.2018.00380)
Supplement: Supplementary file 1 [file Image_1.pdf]

frontiers

# *Supplementary Material*

# Lychee Seed Fraction Inhibits Aβ (1–42)-Induced Neuroinflammation in BV-2 Cells via NF-κB Signaling Pathway


**Ya Zhao [1†], Yuan Zeng [1,2†], Anguo Wu [1†], Chonglin Yu [3], Yong Tang [1], Xiuling Wang [1], Rui Xiong [1], Haixia Chen [1] ,Jianming Wu [1*], and Dalian Qin [1*]**

[1] Laboratory of Chinese Materia Medica, Department of Pharmacology, School of Pharmacy, Southwest Medical University, Luzhou, Sichuan, China

[2] Department of pharmacy, Affiliated Hospital of Southwest Medical University, Luzhou, China,

[3] Department of Human Anatomy, School of Preclinical Medicine, Southwest Medical University, Luzhou, Sichuan, China

*** Correspondence:**
Prof. Jianming Wu
jianmingwu@swmu.edu.cn

Prof. Dalian Qin
dalianqin@swmu.edu.cn


**Supplementary Figures and Tables**

**Supplementary Figure 1.** Identification of the chemical components in LSF using Agilent 6230 UHPLC-DAD-TOF-MS. LSF in methanol was separated on an Agilent Zorbax Eclipse Plus C-18 (100 mm ×2.1 mm) column (particle size: 1.8 μm) at a flow rate of 0.35 mL min$^{-1}$. The data was acquired in the scan mode from m/z 100–1700 Da with 2.0 spectra/s. (A) The total ion chromatogram (TIC) of LSF in negative mode. (B) The total ion chromatogram (TIC) of LSF in positive mode. (C) DAD chromatogram recorded at 280 nm.

**Supplementary Figure 2.** Release of the pro-inflammatory cytokines in Aβ(1-42)-induced BV-2 cells. ELISA was used to determine the levels of IL-1β (A), COX-2 (B) and iNOS (C) in the cell-free supernatants. *$P < 0.05$, **$P < 0.01$, ***$P < 0.001$ vs. Control.

**Supplementary Figure 3.** Inhibition effect of LSF on the release of the pro-inflammatory cytokines in Aβ(1-42)-induced BV-2 cells. BV-2 cells were pretreated with 5 μM Aβ(1-42) for 12 h, then followed with an incubation of 0.007-0.48 mg L$^{-1}$ LSF for another 12 h. ELISA was used to determine the levels of IL-1β (A), COX-2 (B) and iNOS (C) in the cell-free supernatants. **$P < 0.01$ vs. Control; $^{\#}P < 0.05$, $^{\#\#}P < 0.01$, $^{\#\#\#}P < 0.01$ vs. Model.

**Supplementary Figure 4.** Inhibition effect of LSF on the inflammatory cytokines in Aβ(1-42)-induced BV-2 cells. BV-2 cells were pretreated with 5 μM Aβ(1-42) for 24 h, then followed with an incubation

of 0.007-0.48 mg L$^{-1}$ LSF for another 24 h. ELISA was used to determine the levels of IL-1β (A), COX-2 (B) and iNOS (C) in the cell-free supernatants. *$P$ < 0.05, **$P$ < 0.01 vs. Control; $^{\#}P$ < 0.05 vs. Model.

**Supplementary Figure 5.** The full-length Western blotting images.

**Supplementary Figure 1**

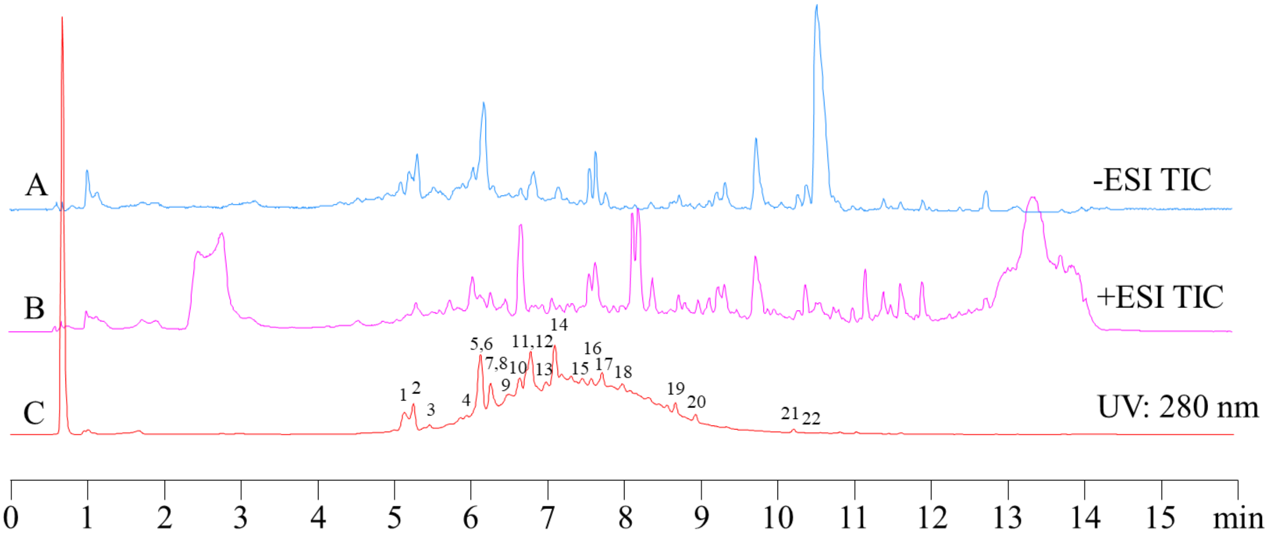

**Supplementary Figure 2**

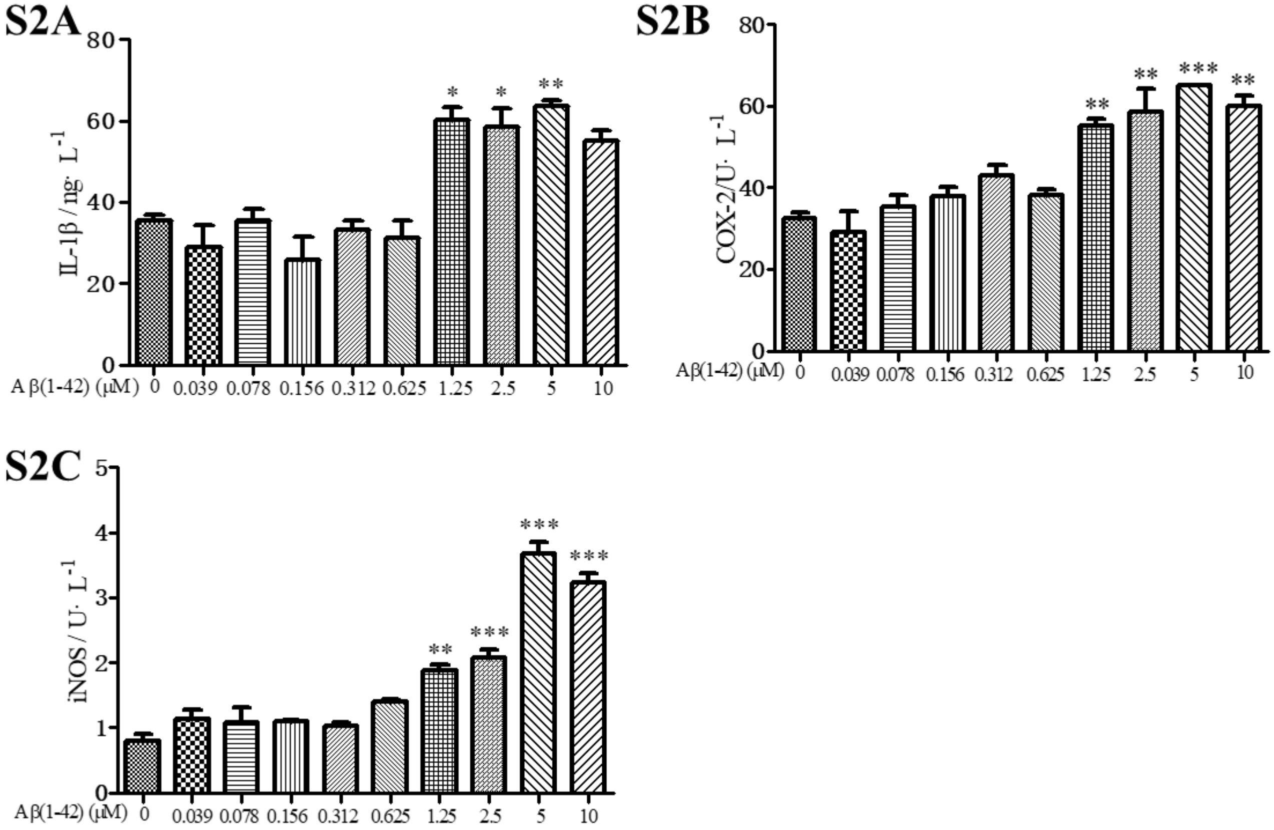

**Supplementary Figure 3**

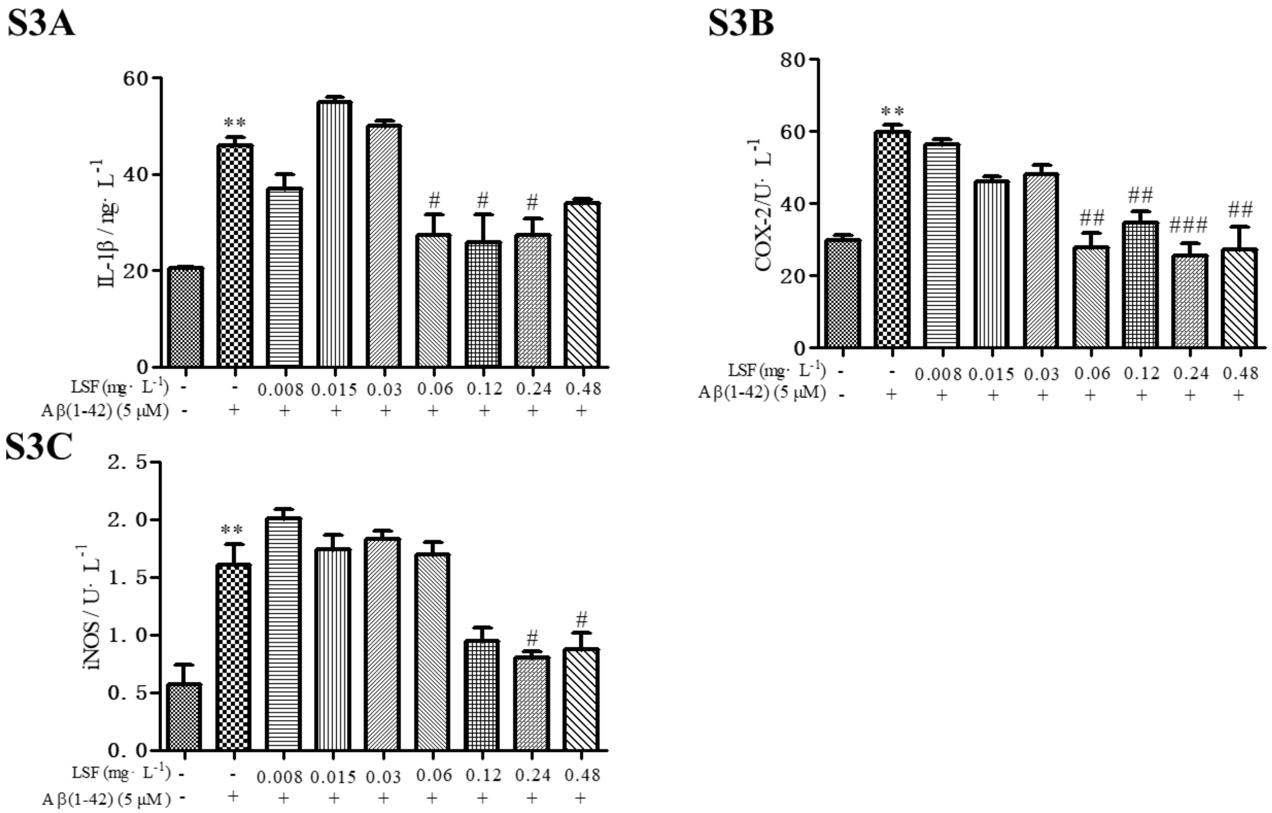

**Supplementary Figure 4**

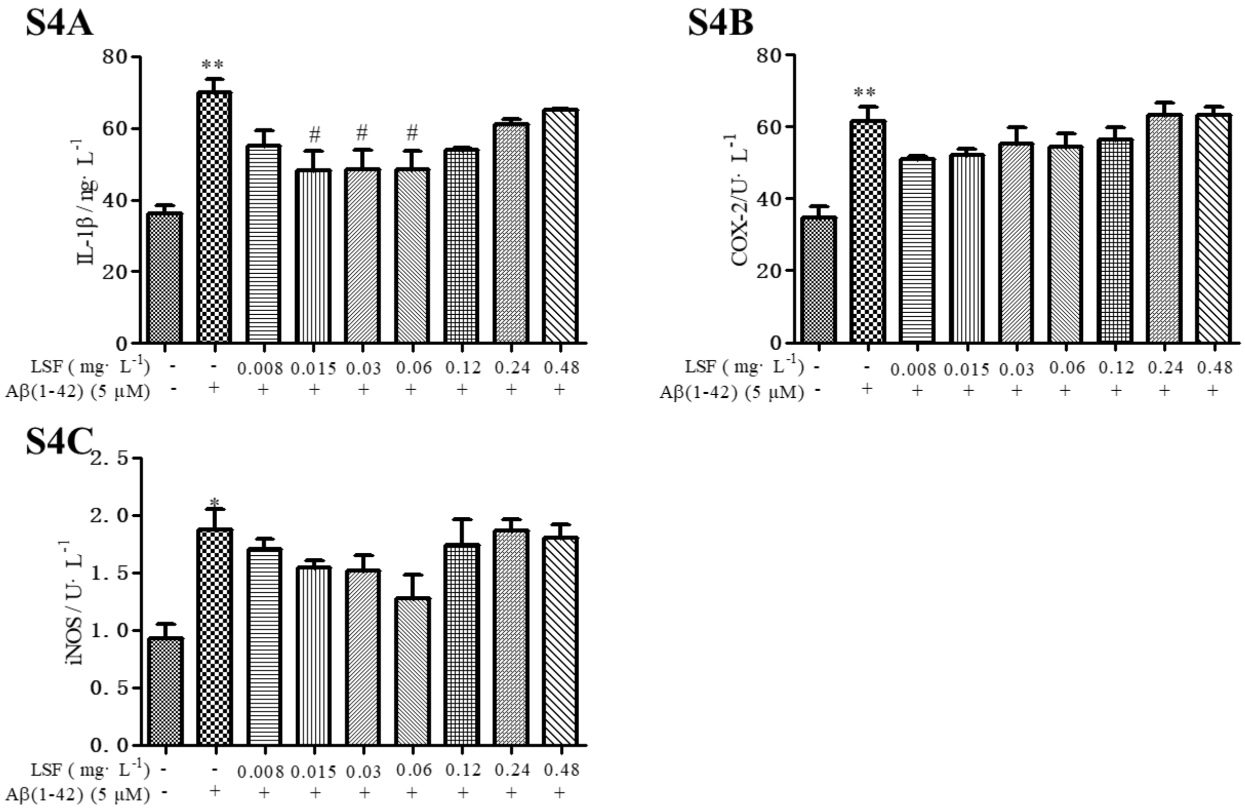

## Supplementary Figure 5

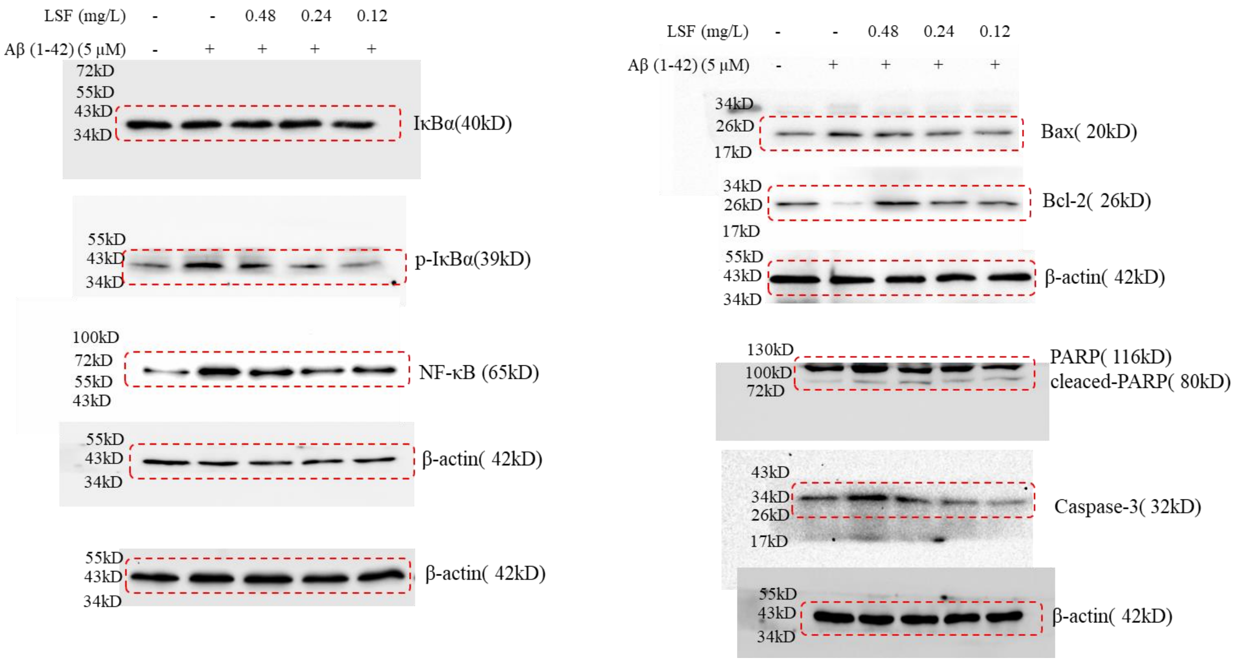

**Supplementary Table 1:** The characterization of the chemical components in LSF by using UHPLC-DAD-TOF-MS analysis in negative and positive mode

| **Peak No.** | **Retention time (min)** | **$[M-H]^-$ (m/z) (Obs)** | **Positive mode** | **(m/z) (Obs)** | **Molecular weight** | **Chemical name** |
|---|---|---|---|---|---|---|
| 1 | 5.262 | 337.0798 | $[M+Na]^+$ | 361.0936 | 338 | Hexahydroxydiphenic acid |
| 2 | 5.347 | 337.0803 | $[M+Na]^+$ | 361.0924 | 338 | Hexahydroxydiphenic acid |
| 3 | 5.578 | 289.0723 | $[M+H]^+$ | 291.0891 | 290 | (–)-Catechin |
| 4 | 5.968 | 577.1175 | $[M+H]^+$ | 579.1541 | 578 | B-type procyanidin trimer |
| 5 | 6.084 | 387.1552 | $[M+Na]^+$ | 411.1666 | 388 | Undentified |
| 6 | 6.167 | 289.0628 | $[M+H]^+$ | 291.0891 | 290 | (–)-Epicatechin |
| 7 | 6.234 | 337.0841 | $[M+Na]^+$ | 361.0926 | 338 | Hexahydroxydiphenic acid |
| 8 | 6.328 | 863.1626 | $[M+H]^+$ | 865.2045 | 864 | A-type procyanidin trimer |
| 9 | 6.535 | 479.1106 | $[M+H]^+$ | 481.2662 | 480 | Unidentified |
| 10 | 6.718 | 863.1626 | $[M+H]^+$ | 865.0246 | 864 | A-type procyanidin trimer |
| 11 | 6.85 | 575.1061 | $[M+H]^+$ | 577.1397 | 576 | A-type procyanidin dimer |
| 12 | 6.89 | 609.1322 | $[M+Na]^+$ | 633.1477 | 610 | Rutin |
| 13 | 7.134 | 463.0794 | $[M+Na]^+$ | 487.2195 | 464 | Quercetin-3-O-β-D-glucoside |
| 14 | 7.214 | 575.1071 | $[M+H]^+$ | 577.1392 | 576 | A-type procyanidin dimer |
| 15 | 7.624 | 419.1833 | $[M+Na]^+$ | 443.1927 | 420 | Unidentified |
| 16 | 7.712 | 405.2047 | $[M+Na]^+$ | 429.2131 | 406 | Litchioside C |
| 17 | 7.815 | 435.1204 | $[M+Na]^+$ | 459.1294 | 436 | Phlorizin |
| 18 | 8.048 | 367.0619 | $[M+Na]^+$ | 367.2116 | 368 | Unidentified |
| 19 | 8.779 | 283.055 | $[M+Na]^+$ | 307.0599 | 284 | Unidentified |
| 20 | 9.05 | 583.3368 | $[M+Na]^+$ | 607.3498 | 584 | Litchioside A |
| 21 | 10.105 | 865.3929 | $[M+H]^+$ | 867.378 | 866 | B-type procyanidin trimer |
| 22 | 10.324 | 329.2328 | $[M+Na]^+$ | 353.2308 | 330 | Unidentified |
